# One case of recurrent ST-segment elevation myocardial infarction in a patient with antiphospholipid syndrome complicated with severe thrombocytopenia within a short period of time: A case report

**DOI:** 10.1097/MD.0000000000035775

**Published:** 2023-11-10

**Authors:** Zhongjue Qiu, Yong Wang, Li Xu, Zhou Zhou, Jiacheng Zhang, Zhen Wang

**Affiliations:** a First Clinical Medical College, Shandong University of Traditional Chinese Medicine, Jinan, China; b Department of Cardiology, Affiliated Hospital of Shandong University of Traditional Chinese Medicine, Jinan, China; c Center for post-doctoral studies, Shandong University of Traditional Chinese Medicine, Jinan, China; d School of Foreign Languages, Shandong University of Traditional Chinese Medicine, Jinan, China.

**Keywords:** anticoagulant regimen, antiphospholipid syndrome, severe thrombocytopenia, ST-segment elevation myocardial infarction

## Abstract

**Introduction:**

Acute myocardial infarction is an important arterial thrombotic event in patients with antiphospholipid syndrome (APS). Anticoagulation regimen might greatly affect the prognosis. Due to the lack of sufficient data and large prospective randomized controlled trials, there is no definite consensus among researchers on the optimal antithrombotic therapy for patients with APS after arterial events.

**Patient concerns:**

A 35-year-old male presented with sustained chest tightness and chest pain for 6 hours, accompanied with palpitation, sweating and headache.

**Diagnosis:**

The patients was diagnosed with acute ST-segment elevation myocardial infarctions with severe thrombocytopenia. Emergency coronary angiography showed that the posterior descending artery (PDA) was occluded, and a thrombus shadow was visible. An emergency coronary intervention was performed to open the occluded PDA. About 48 hours after hospitalization, the PDA was occluded again and percutaneous coronary intervention was performed again to open the blocked coronary artery. The lupus anticoagulant screen rate were positive during hospitalization and 12 weeks after discharge, meeting the diagnostic criteria of APS.

**Interventions:**

The patient received coronary intervention twice during hospitalization. After the second coronary intervention, a sequential therapy of bivalirudin, fondaparinux sodium, and warfarin was adopted as the anticoagulant regimen.

**Outcomes:**

The patient was discharged in stable condition without chest pain. One year later, during her follow-up, a repeat echocardiogram and electrocardiogram showed normal findings.

**Conclusion:**

It is the first report of severe thrombocytopenia and recurrent coronary thrombosis within a short period of time in an APS patient, and in this report the importance of anticoagulant therapy in thrombus management in patients with APS was present, also the importance of tracking thrombotic factors. This study proposes an anticoagulation regimen for patients suffering from antiphospholipid syndrome, experiencing recurrent atherothrombotic events, and presenting severe thrombocytopenia.

## 1. Introduction

Antiphospholipid syndrome (APS), also known as antiphospholipid antibody syndrome, is a systemic autoimmune disorder characterized by thrombosis caused by antiphospholipid antibodies (mainly ACA, lupus anticoagulants [LA], and anti-beta 2 glycoprotein I). In this case, recurrent acute coronary thrombosis events occurred consecutively within 3 days, in which 1 event occurred when anticoagulant drugs were stopped due to severe thrombocytopenia with antiplatelet therapy applied alone, indicated the importance of anticoagulant therapy in the treatment of arterial thrombotic events in APS patients. Therefore, anticoagulation regimen might greatly affect the prognosis. For acute myocardial infarction patients with APS and severe thrombocytopenia, perioperative sequential therapy with bivalirudin, fondaparinux sodium, and warfarin might be a safe and effective anticoagulant regimen. Patient has provided informed consent for publication of the case.

## 2. Case presentation

**Figure 1.**
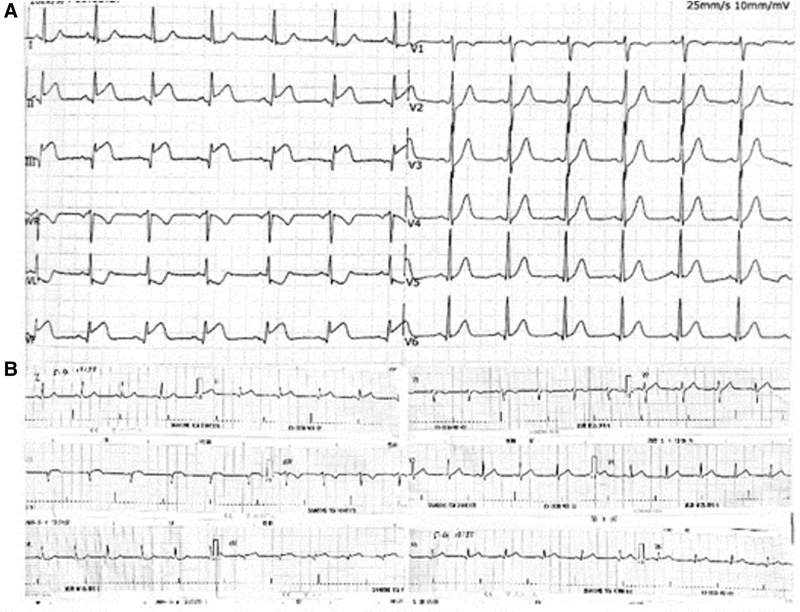
(A) Electrocardiogram during the first ACS ECG shows ST-segment elevation in leads II, III and aVF. (B) ST-segment regression in leads II, III and aVF after PCI. ECG = electrocardiogram, PCI = percutaneous coronary intervention.

**Figure 2.**
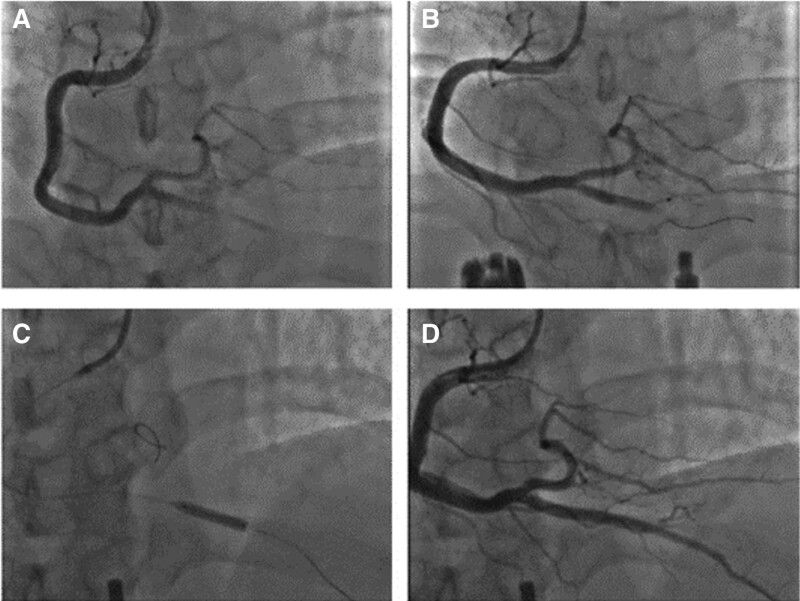
Right coronary artery. (A) Complete occlusion of the posterior descending branch of the right coronary artery. (B) Passage of the guidewire through the occluded segment of the lesion. (C) Repeated pre-expansion balloon dilation followed by implantation of 2 stents. (D) Posterior descending branch angiography after stenting.

A 35-year-old male was admitted to the hospital due to sustained chest tightness and chest pain for 6 hours, accompanied with palpitation, sweating and headache. One month ago, due to traumatic trauma, he underwent open reduction and internal fixation for lumbar fractures in our hospital. At that time, the platelet count was 114 × 10^9^/L. The patient had no history of hypertension, dyslipidemia, diabetes or coronary heart disease, and no cardiovascular risk factor except for smoking about 1 to 2 cigarettes per day. On admission, he showed normal vital signs, with electrocardiogram (ECG) showing ST-segment elevation in leads II, III, and aVF, and therefore the diagnosis was acute ST-segment elevation myocardial infarction (STEMI) (Fig. 1). Emergency coronary angiography showed that the posterior descending artery of the right coronary artery was occluded, and a thrombus shadow was visible. The following interventional treatment was conducted: The guidewire was sent to distal end of the posterior descending branch, and then the balloon dilation catheter were sent to the lesion position. After that balloons were dilated repeatedly, and the 2.5 × 24 and 3.0 × 14 mm stents were implanted in tandem. Repeated angiography showed that the blood vessels were unobstructed and there was no residual stenosis (Fig. 2). Chest pain was relieved postoperatively.

Heparin was used for anticoagulation during the operation, and aspirin, ticagrelor, and tirofiban were administered as the antithrombotic therapy after the operation. The postoperative platelet count was 43 × 10^9^/L, and the D-dimer was 6.50 µg/mL. The platelet and D-dimer were checked within 24 hours repeatedly and the results showed a downward trend in platelet count gradually, finally down to 33 × 10^9^/L, while a upward trend in D-dimer test gradually, and finally up to 16.35 µg/mL. Given the high bleeding risk, the administration of tirofiban was discontinued.

**Figure 3.**
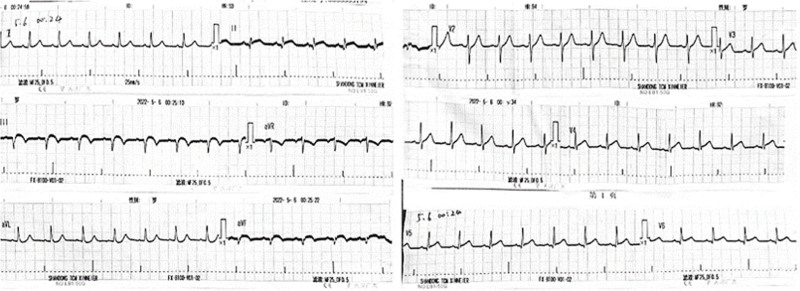
ECG during an in-stent thrombotic event. Compared to the ECG after first PCI, the ECG shows renewed ST-segment elevation in the leads II, III and aVF. ECG = electrocardiogram, PCI = percutaneous coronary intervention.

**Figure 4.**
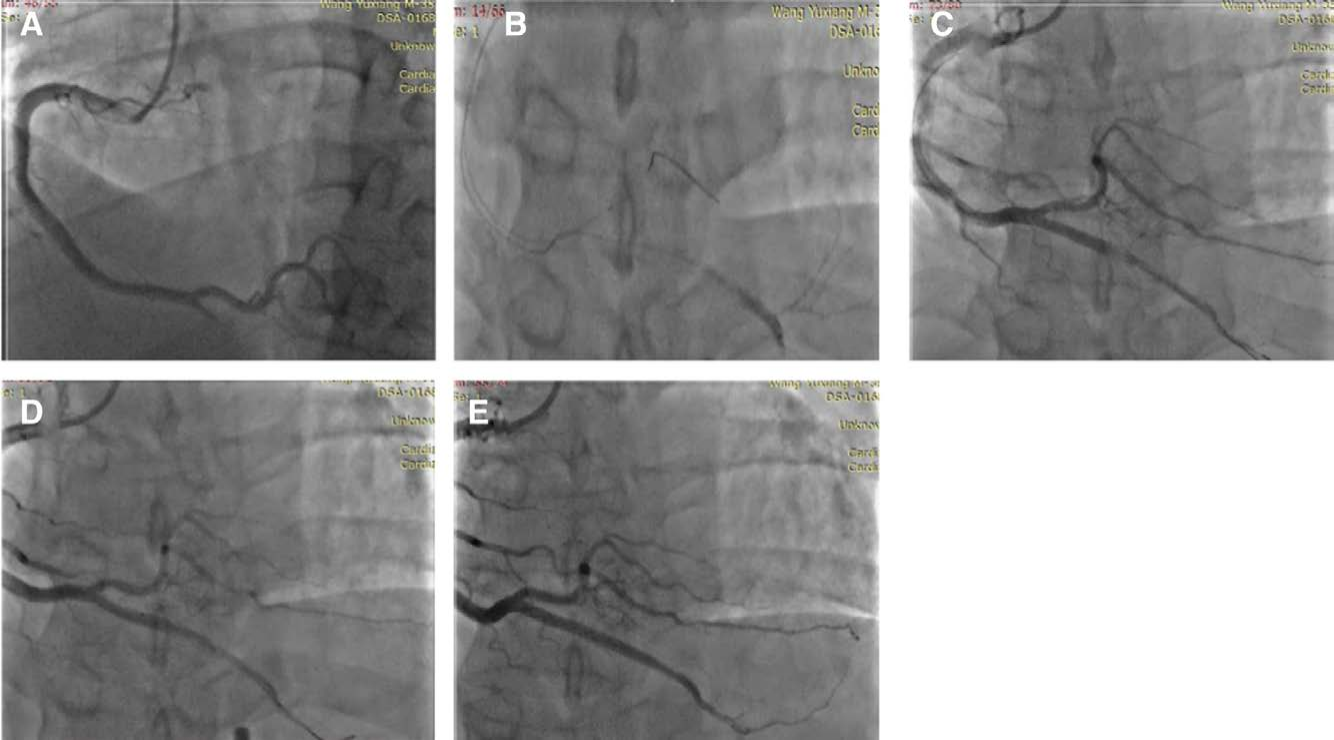
Right coronary artery. (A) Complete occlusion of the distal posterior descending branch of the RCA with a thrombotic shadow visible the stent. (B) Delivery of PILOT 50 guide wire with microcatheter support to the distal part of the occluded segment. (C) Stent proximal thrombus shadow and distal dissection. (D) Intra-coronary thrombus aspiration and intracoronary stenting for coronary artery dissection. (E) Posterior descending branch angiography after treatment. RCA = right coronary artery.

About 48 hour after hospitalization, in the patient there was the recurrence of unrelieved chest pain, and ECG showed ST-segment elevation in leads II, III, and AVF compared with the ECG in the postoperative period (Fig. 3). Coronary angiography performed in emergency again showed that the distal end of right coronary artery posterior descending artery was completely occluded again and the thrombus load was high. The guidewire was sent to the posterior descending branch, but repeated dilation of balloon and thrombus suction had little effect. Angiography showed a thrombus shadow in the proximal of the stent and a dissection in the distal. A 2.25 × 20 mm stent was implanted at the dissection, and the thrombus was squeezed into the distal end by repeated dilation of balloon. Finally, angiography showed that the blood flow in the posterior descending artery was restored, but the blood flow was of grade II of thrombolysis in myocardial infarction risk score and the thrombus shadow was visible in the distal end (Fig. 4). The platelet count significantly decreased with high risk of bleeding, and therefore bivalirudin was administered for anticoagulation during the operation.

Repeated laboratory test showed that platelet count decreased to 26 × 10^9^/L, indicating a paradox in the treatment, the platelet count decreasing sharply with high risk of bleeding while the recurrence of acute myocardial infarction in a short period of time with high thrombosis load. Bivalirudin, aspirin and ticagrelor were administered for antithrombotic therapy, and at the same time, the causes of thrombocytopenia and high thrombus load were identified. Laboratory results were as follows: Lupus anticoagulant screen rate was 3.06 (>1.2, positive), ACA level was negative, antinuclear antibody level was negative, immunoglobulin level was negative, and anti-beta 2 glycoprotein I level was negative. Therefore, the patient was suspected of suffering from APS and methylprednisolone was administered. Although the instruction of bivalirudin states that it could not be administered more than 24 hours after intervention, given the relatively low risk of bleeding, bivalirudin was administered continuously for 50 hours after surgery, and the platelet count increased to 56 × 10^9^/L. After that, aspirin and ticagrelor combined with fondaparinux sodium were administered to replace bivalirudin for antithrombotic therapy. The platelet count increased to 117 × 10^9^/L. Three days after the intervention, warfarin was also administered. Three days after the combined intervention, the antithrombotic regimen was changed again and was composed of aspirin, Plavix, and warfarin. After discharge from hospital, the patient continued to take warfarin, aspirin, and Plavix. The 3-month follow-up showed there was no recurrence of thrombosis, and lupus anticoagulant screen rate was 1.33 (>1.2, positive), meeting the diagnostic criteria of APS. One year later, during his follow-up, this patient recovered well, and there was no chest tightness and chest pain after activities, and color Doppler echocardiogram showed normal cardiac function without abnormal ventricular wall motion. The patient was advised to continue treatment and attend regular cardiac and rheumatic follow-up examinations.

## 3. Discussion and conclusion

APS is a condition caused by antiphospholipid antibodies (ACA, LA, and anti-B2GPI), and is a systemic autoimmune disease mainly characterized by thrombosis. Thrombosis mainly includes the following aspects: venous system thrombosis with deep vein thrombosis as the major manifestation; arterial thrombosis with stroke and myocardial infarction as the major manifestation; microvascular thrombosis with catastrophic antiphospholipid syndrome as the major manifestation. Nonthrombotic (atypical) manifestations of APS include cholecystitis and longitudinal myelitis, inflammatory or thrombotic (with fibrin deposition) valvulitis, and thrombocytopenia. The patient was diagnosed with APS presenting with severe thrombocytopenia and recurrent myocardial infarctions. Dual antiplatelet therapy of aspirin and clopidogrel sulfate was used continuously after coronary interventional therapy. The use of anticoagulant drugs was gradually modified with the recovery of platelet count and the extension of treatment time. Postoperatively the patient exhibited severe thrombocytopenia with thrombocytopenia to 26 × 10^9/L, bivalirudin was continuously administered to 50 hours. Upon satisfactory elevation of the patient’s platelet count (to 56 × 10^9^/L), fondaparinux sodium was administered as an alternative to bivalirudin. As the platelet count returned to ordinary levels, the anticoagulant therapy was revised wherein fondaparinux sodium and warfarin were simultaneously used for a period of 3 days. Following this, the usage of fondaparinux sodium was halted and aspirin, clopidogrel, and warfarin were introduced into the antithrombotic treatment regimen. Post-discharge, administration of warfarin, aspirin, and clopidogrel remained consistent in the patient’s treatment protocol.

Laboratory examination for APS consists of test for ACA, LA, and anti-B2GI, and the diagnostic criteria are at least 1 clinical manifestation (thrombotic event or pregnancy complication) and at least 1 positive result of laboratory examination (at least 2 positives results of the following tests per 12 weeks: medium-high titer IgG/IgM ACA, IgG/IgM anti-β2GI antibody, LA). In this case, a recurrent coronary thrombotic event occurred within a short period of time with the presence of clinical symptoms. LA levels increased to 3.06, was still positive after 12 weeks (1.33), meeting the laboratory diagnostic criteria. Therefore, APS was definitely diagnosed in this patient.

Since antiphospholipid antibody (APLA) positivity will affect treatment choice, APLA test should be performed in the following conditions: arterial events and complicated with rheumatic or autoimmune diseases^[1]^; patients less than 50 years old with arterial events but without documented risk factors^[2]^; and cryptogenic stroke and myocardial infarction with non-obstructive coronary artery.^[3]^ Similar to recurrent coronary thrombotic events previously reported,^[4]^ in this case the importance of diagnosing the potential thrombotic disorder was emphasized, and the diagnosis and test by us were more promptly. Due to the lack of sufficient data and large prospective randomized controlled trials, there is no definite consensus among researchers on the optimal antithrombotic therapy for patients with APS after arterial events.

According to some reports, warfarin is more effective in preventing arterial thrombosis (recurrence rate: 1.05/100 patients/yr) than venous thrombosis (recurrence rate: 10.53/100 patients/yr), possibly influenced by the formation of thrombus.^[5]^ According to a retrospective cohort study by Ohnishi et al^[6]^ in 2018, warfarin combined with antiplatelet therapy is effective for preventing the recurrence of arterial thrombosis in patients with high risk of APS, and this combination is better than warfarin monotherapy. Currently, many studies suggested that patients should receive warfarin with an international normalized ratio >3.0 or low-dose aspirin plus standard-strength warfarin with international normalized ratio 2 to 3. A study showed that on the secondary prevention of ischemic stroke in APS patients, although the risk of bleeding complications was similar, compared with antiplatelet therapy alone, combined antiplatelet and anticoagulant therapy was of lower incidence of stroke.^[7]^ Another retrospective study showed that combined antiplatelet and anticoagulant therapy could reduce the recurrence rate of thrombosis in patients with APS and arterial thrombosis.^[8]^

Since the stent is metallic, there is a high probability of stent thrombosis after percutaneous coronary intervention (PCI). For APS patients who must be implanted with stents, a short-term combination of dual antiplatelet therapy and warfarin is recommended.^[9,10]^ For APS patients receiving combined dual antiplatelet therapy and warfarin after PCI, according to some cases reported, the duration of dual antiplatelet therapy ranged from 1 to 12 months (Plavix was recommended), and the overall prognosis was good. Except for the white thrombus reported by Rocco Vergallo after discontinuing clopidogrel and repeated stent thrombosis events leading to the coronary artery bypass surgery reported by Li Hong, very few patients experienced recurrence of coronary artery thrombosis^[11,12]^

For this patient, because the diagnosis of APS was not clear during the first STEMI, when the platelet count of this patient decreased to 26 × 10^9^/L, considering the high bleeding risk, we discontinued tirofiban. Thrombotic events still occurred despite dual antiplatelet therapy, indicating the importance of anticoagulant therapy in the management of thrombus in APS after PCI. After APLA positivity was determined, we adopted a triple antithrombotic regimen of dual antiplatelet therapy plus another anticoagulant. Since bivalirudin could be used no more than 24 hours postoperatively and warfarin took effect 3 days after surgery, we urgently needed an anticoagulant to bridge the gap. Although it has been reported that most patients were given anticoagulant of heparin with low molecular weight after stent thrombosis, considering the severe thrombocytopenia in patients and the low bleeding risk of fondaparinux sodium compared with heparin, we finally chose fondaparinux sodium. Afterwards, fondaparinux sodium was replaced by warfarin. Finally, Plavix was administered instead of ticagrelor, combined with aspirin and warfarin to form a triple antithrombotic therapy regimen, and the patient recovered well after the treatment. The patient did not undergo coronary angiography during the follow-up period for personal reasons and therefore did not provide us with angiographic images after 1 year of prognosis. This limitation prevented us from investigating the patient’s vascular status in detail.

In addition to the hypercoagulability, arterial thrombosis in APS is also related to arteriosclerosis.^[13,14]^ More and more studies have shown that APS could induce the occurrence of atherosclerosis. Since oxidized low density lipoprotein is involved in the pathophysiology of atherosclerosis, statin in the primary prevention should not be overlooked. In addition to lipid-lowering effects, other potential effects, especially anti-inflammatory and antithrombotic effects, are also verified by in vitro experiments and in vitro studies.^[15,16]^ We also use statins in patients to prevent coronary atherosclerosis.

In conclusion, we reported the treatment of recurrent STEMI in an APS patient complicated with severe thrombocytopenia. It is the first report of severe thrombocytopenia and recurrent coronary thrombosis within a short period of time in an APS patient, and in this report the importance of anticoagulant therapy in thrombus management in patients with APS was present, also the importance of tracking thrombotic factors. Our postoperative anticoagulant therapy was a sequential therapy consisting of bivalirudin, fondaparinux sodium, and warfarin, and we hope this study could provide a reference for the anticoagulant therapy in APS patients with thrombocytopenia after PCI.

## Author contributions

**Data curation:** Zhongjue Qiu, Yong Wang, Zhen Wang.

**Investigation:** Yong Wang, Jiacheng Zhang, Zhen Wang.

**Methodology:** Zhongjue Qiu, Yong Wang, Zhen Wang.

**Writing – original draft:** Zhongjue Qiu, Yong Wang, Li Xu.

**Writing – review & editing:** Zhou Zhou, Zhen Wang.
